# Potential propensity of traditional herbal materials ingested by collegiate athletes in South China for their consuming health care

**DOI:** 10.1097/MD.0000000000027635

**Published:** 2021-11-05

**Authors:** Yue-Quan Qin, Chun-Song Cheng, Ying Jiang, Wei Qi, Bin Zhang, Dong-Yi Wei

**Affiliations:** a Institute of Physical Education, Guangxi University of Chinese Medicine, Nanning, China; b Ganjiang Institute of Medicinal Plants, Lushan Botanical Garden, Chinese Academy of Sciences, Jiujiang, China; c Macau University of Science and Technology, Taipa, Macao, China; d Sports Teaching and Research Room, Anhui University of Traditional Chinese Medicine, Hefei, China; e Institute of Physical Education, Anhui Normal University, Wuhu, China.

**Keywords:** cardiovascular receptors, herbal tea, plant-derived stimulants, slow-cooked soup, sports doping

## Abstract

In south China, traditional herbal medicines have been widely used as functional foods or dietary supplements for daily health care. Many plant-derived chemical substances with biological activity are inadvertently ingested by collegiate athletes daily through canton-style herbal tea or herbal slow-cooked soup. In the view of the complexity of herbal ingredients, it is still no full survey reported for the sports risk of plant-derived sports doping. This research is firstly a descriptive statistical analysis. Collegiate athletes with different socio-economic characteristics from medical colleges in 3 different regions in China participated in the questionnaire survey. Three survey forms, including the oral interview, email inquiry, handing out and recovering the questionnaires in live, were developed and performed by researchers. It was first found that collegiate athletes resorted to some traditional herbal materials to protect their health care that there were regional differences (*P* < .01). Collegiate athletes with Health Fitness and Traditional Wushu as their sports expertise showed a higher frequency of recognition or ingestion in the use of traditional herbal materials (*P* < .01), while their different living types and cuisine preferences did not seem to be associated with the ingestion frequency of traditional herbal materials. In addition, in the view of the significant differences in the use of herbal preparations to relieve sports stress among young athletes in different regions (*P* < .01), the findings strongly suggested that athletes should strictly control their use of various herbal preparations during sports training and competition, including herbal wines, herbal oils, topical plasters, analgesic tablets.

## 1 Introduction

In China, a large portion of traditional herbal medicines have been widely used as functional food or dietary supplement for daily health care. Although rare in most parts of the world, health care consumption of canton-style herbal tea and herbal slow-cooked soup has always been very popular in south China,^[1]^ especially in Guangdong and Guangxi provinces. It is generally known that the most herbs are claimed to have been used safely for many centuries. However, a large number of herbal medicines are still used without rigorous safety assessment. Scientists around the world have still not given up on exploring and monitoring the potential relationship between herbal consumption and the safety for specific consumer populations.^[2–4]^ In recent years, more and more chemical substances and conditions have been concerned and included in the list of sports stimulants.^[5]^ The inclusive principle of the list of doping in sports is entirely based on the mechanism of modern pharmacology and biomedicines. All medicines with potential impact on athletes’ sports performance should be included in the category of banned substances. Therefore, the sports risk from the consumption of herbal tea and herbal slow-cooked soup, needs to be carefully evaluated for the vast number of athletes in South China. In recent years, many plant-derived substances, such as the ephedrine, noraconitine, morphine, etc,^[6]^ had been successively listed in the International Convention Against Doping in Sports^[7]^ and prohibited from being used in daily training and sports competition to avoid interfering with sports performance.

It is worth noting that the China Anti-Doping Agency issued a special document on the potential risks of β_2_ receptor agonist in November 2016, which directly involved 7 categories of plants and 2 kinds of plant- derived ingredients that are threatened. Although the scope of the list published by International Convention Against Doping in Sports covers a wide range in Chinese daily consumption, in view of the complexity of herbal ingredients, it is still not a full survey for the sports risk of herbal consumption. In addition to the β_2_ receptor agonists, the list of banned drugs also involves other plant derived stimulants, narcotics, as well as the dangerous ingredients from medicinal marijuana.

This investigation was conducted to figure out the use of traditional herbal materials ingested for daily health care by collegiate athletes with different educational backgrounds in Guangdong and Guangxi provinces. This study was also conducted to analyze the potential risks in their sports performance, which may come from the consumption of canton-style herbal tea and herbal slow-cooked soup.

## 2 Methods

### 2.1 Design

This investigation includes a descriptive analysis of the consumption of herbal materials, which may constitute sports risk to a certain extent, and a cross-sectional study on college athletes from different educational backgrounds in Guangdong and Guangxi provinces.

### 2.2 Setting and sampling

This study was conducted in 3 different universities in Guangdong and Guangxi provinces and the Macao Special Administrative Region, including Guangzhou University of Traditional Chinese Medicine, and Guangxi University of Traditional Chinese Medicine. As a control group, we also served the same survey in a university of central China, it is Anhui University of Traditional Chinese Medicine. Due to the lack of human resources during the investigation, we did not choose other comprehensive universities as the survey sites. Besides, during our preliminary preparations, we learned that students in colleges of traditional Chinese medicine are significantly sensitive to the common herbal materials than other collegiate students without medicinal backgrounds. So, we randomly surveyed more than 10 different disciplines in the mentioned 3 universities, which were divided into 3 groups in the follow description according to their academic characters defined as general education (GE), clinical medicine (CM), and pharmaceutical majors (PM). Conventionally, GE usually includes literature, physical education, linguistics, law, history, management and other majors; CM usually refers to all clinical specialties, including clinical medicine, clinical medicine in traditional Chinese medicine as well as the integrated clinical medicine; PM includes all majors in pharmacy, pharmaceutical engineering, traditional Chinese medicines, medicinal botany, pharmacy, etc. The valid sample that covers all the positive respondents in this study consisted of a total of 339 collegiate athletes who participate in daily sports training and irregular special sports competitions.

### 2.3 Management and measurement

There are 3 survey forms, including the oral interview (approximately 30 minutes per person), sending and receiving questionnaires with email (Data collection took an average of 20 minutes for each collegiate athletes), handing out and recovering the questionnaires in live. We stated at the beginning of all interviews or marked out with a statement at the beginning of the questionnaire that personal information should always be protected during and after this survey. We also beautifully collaborated a brochure for the interviewees before the investigation started, which is covering nearly 100 popular canton-style herbal teas and herbal slow-cooked soup with colorful pictures. The data in this survey were collected through an electronic questionnaire form developed by our research team.

The questionnaire form covers 5 separate parts and includs a total of 27 questions about the socio-demographic characteristics of the interviewed collegiate athletes (with 6 questions about their basic information); scale systems of physical diathesis and exercise intensities (12 questions); frequently-used dietary supplements for improving physical diathesis (10 questions); eating habits during daily training, pressurized training session and competitive period (6 questions); Differences in dietary supplements at home and college during non-training period. The questionnaires were answered by the athletes in person. Data collection took an average of 20 to 25 minutes for each student.

### 2.4 Data collection and analysis

The data obtained and recorded with Excel 2014, and then analyzed with Chi square (Pearson Chi-Squared, 2x^2^ comparison was conducted for association between 2 categorical variables), *t*-test (only for repeated test comparison). The simple percentage and arithmetic mean were conducted in SPSS 16.0 program. R-Studio with R version 3.3.3 and ggplot 2 package was used for the graphical representation of the data.

### 2.5 Ethical aspect of the study

The study was sponsored by the special fund for talents in Guangxi University of Traditional Chinese Medicine (2018MA050) and the Key Project of Humanities and Social Science Research in Anhui Province (SK2020A0252), approved by the Sports Teaching Department of the Guangxi University of Traditional Chinese Medicine and Anhui University of Traditional Chinese Medicine (NO.: GXUCM-IRB-H2). Because, managing the diet of collegiate athletes is one of the important responsibilities in the conventional college physical education, further ethical review was exempted by the IRB of Guangxi University of Traditional Chinese Medicine. This study only carried out surveys and statistics on the functional food materials ingested by collegiate athletes daily, and initially discussed the possible propensity of food in the context of customs in different regions, and did not take any measures to investigate the direct biochemical indicators or behaviors of athletes. In order to be able to collect accurate information, it was explained in the consent paper from that it was not obligatory to write real name on the consent form and all information would remain anonymous in this study.

## 3 Results

### 3.1 The analysis of athlete's socio-economic characteristics

The collegiate athlete's socio-economic characteristics were first analyzed, and the results showed as following Table 1 that 47.8% of the total questioned students were between 20 to 24 years old, and the ratio of male and female athletes is close to 1.0:1.4. All respondents in this study indicated that they had frequent ingestion of traditional herbal materials, and nearly 70% of them came from the South China, the 13% of them came from the North China, and the remaining 11% of them came from the East China. We also found that collegiate athletes majoring in CM and PM, 46.9% and 39.5% of the respondents respectively, were more inclined to accept traditional herbs as their dietary supplements. Students with *health fitness* and *traditional wushu* as their sports expertise showed higher frequency of recognition or ingestion in the use of traditional herbal materials. Besides, as shown in this result, the different living types and cuisine preferences did not seem to be associated with the ingestion frequency of traditional herbal materials.

**Table 1:** The collegiate athlete's socio-economic characteristics (N = 339).

	Male	Female	Total
	n1 (%)	n2 (%)	N (%)
Age (yr)			
<20	41 (27.3)	56 (29.6)	97 (28.6)
20–24	76 (50.7)	86 (45.5)	162 (47.8)
> 24	33 (22.0)	47 (24.9)	80 (23.6)
Hometown			
North China	31 (19.3)	13 (7.3)	44 (13.0)
South China	102 (63.3)	134 (75.3)	236 (69.6)
East China	15 (9.3)	23 (12.9)	38 (11.2)
Southwest of China	13 (8.1)	8 (4.5)	21 (6.2)
Academic characters^#^			
GE	29 (16.3)	17 (10.6)	46 (13.6)
CM	81 (45.5)	78 (48.4)	159 (46.9)
PM	68 (38.2)	66 (41.0)	134 (39.5)
Sports expertise			
Traditional Wushu^∗^	77 (45.0)	58 (34.5)	135 (39.8)
Health fitness^∗^	80 (46.8)	80 (47.6)	160 (67.2)
Rhythmic gymnastics	5 (2.9)	28 (16.7)	33 (9.7)
Athletics	9 (5.3)	2 (1.2)	11 (3.3)
Living type			
Village	54 (35.5)	35 (18.7)	89 (26.3)
Metropolis	48 (31.6)	63 (33.7)	111 (32.7)
Townlet	50 (32.9)	89 (47.6)	139 (41.0)
Chinese cuisine preferences			
Canton cuisine	43 (28.1)	62 (33.3)	105 (31.0)
Sichuan cuisine	52 (34.0)	46 (24.7)	98 (29.0)
Hunan cuisine	36 (23.5)	38 (20.4)	74 (21.8)
Shanghai cuisine	9 (5.9)	23 (12.4)	32 (9.4)
Other cuisines	13 (8.5)	17 (9.2)	30 (8.8)

# General education (GE), clinical medicine (CM), and pharmaceutical majors (PM). ^∗∗^Pearson Chi-Squared, *P* < .01.

### 3.2 The purposes of collegiate athletes using the traditional herbal materials

The most of the collegiate athletes were found to resort to the traditional herbal materials to increase the nutrients, energy, and food diversity, or improve their sleeping quality, gastrointestinal function, circulation, or promote the metabolism, anti-inflammatory, or relieve the physical stress, or assist in the physical recovery. In this study, an interesting result in Table 2 was showed that athletes with high exercise intensity, both male and female, are dependent more on traditional herbs for their physical recovery (Pearson Chi-Squared, *P* < .05). It also seems that many athletes with weak physical constitution are found to resort to the traditional herbal materials to improve their sleeping quality, but the analysis results did not support this among in the collegiate athletes (Pearson Chi-Squared, *P* > .05). The current survey also showed that collegiate athletes’ main purposes of using the traditional herbal materials include promoting the physical recovery (98.82%) and metabolism (73.75%), increasing the food diversity (99.12%), nutrients (77.88%) and promoting their energy (83.19%). We were also concerned about some obvious benefits of intaking herbal medicine, which seems not widely accepted by athletes in our study here, such as improving sleep quality (11.5%), relieving physical stress (3.5%), as well as promoting anti-inflammatory (3.8%).

**Table 2:** The purposes of collegiate athletes using the traditional herbal materials.

	Scale score of physical consritution					Scale score of exercise intensities				
	<8		8–10			<8		8–10		
Purposes of using the traditional herbal materials	Male	Female	Male	Female	Test (P)	Male	Female	Male	Female	Test (P)
To improve sleep quality#	14	18	2	5	0.46	10	12	6	11	0.52
to improve gastrointestinal function	12	17	15	16	0.59	13	18	14	15	0.62
To increase the nutrients ^	54	79	50	81	0.69	51	77	53	83	0.89
To improve the circulation	6	33	5	23	0.79	4	31	7	25	0.25
To relieve the physical stress!	3	5	1	3	0.67	2	1	2	7	0.16
To promote metabolism ^	64	52	66	68	0.35	62	61	68	73	0.72
To promote anti-inflammatory!	2	5	2	4	0.85	3	5	1	4	0.51
Assist in the physical recovery ^^	83	122	59	71	0.38	61	63	81	135	**0.04** ^∗^
To increase energy ^	62	70	65	85	0.54	42	74	72	87	0.13
To increase the food diversity ^^	69	107	72	88	0.28	67	76	74	119	0.12
Other therapeutic purposes	14	32	8	37	0.16	11	39	11	30	0.59

∗ Pearson Chi-Squared was used for test, *P* < .05.

### 3.3 Topical herbal preparation or acupressure used to relieve the pain and body stress

Young athletes do not have a huge demand for supplementary foods or herbal medicines in terms of their sleep management and anti-inflammatory due to their well-prepared physical fitness. However, in the face of the huge demand of relieving physical stress after their intensive physical training, they do not ask for suitable herbal slow-cooked soup. This is a very interesting survey result and there are probably strong alternatives to relieve their physical stress better. We did an investigation in-depth on how to relieve the body stress after a high-intensity exercise. As shown in the following Table 3, young athletes tend to use topical herbal preparations or traditional acupressures to relieve the pain and body stress caused by the lactic acid accumulation or muscle fatigue. There are significant differences in the preference for herbal wine, external plaster, analgesic tablet, and the acupoint pressure among collegiate athletes from the 3 universities in different places and different cultures (Pearson Chi-Squared, *P* < .01 or *P* < .001). Indeed, collegiate athletes from different places have a similar preference for using the external herbal oils. There are many kinds of external preparations of Chinese herbal medicines, and they are widely distributed in the Greater China markets and are very easy to gain access to use by ordinary people. Moreover, the diversified types of herbal medicines with complex sources in numerous topical preparations, especially highly toxic ones, exacerbate the risk of stimulant intake for athletes.

**Table 3:** topical herbal preparations or acupressure used to relieve the pain and body stress.

	Guangdong		Guangxi		Anhui		Test
Areas alternatives	Have used	Have not	Have used	Have not	Have used	Have not	*P* value
Herbal wine	18 (15.0)	102 (85.0)	26 (20.3)	102 (79.7)	30 (33.0)	61 (67.0)	.007^∗^
Herbal oil	54 (45.0)	66 (55.0)	61 (47.7)	67 (52.3)	55 (60.4)	36 (39.6)	.068
Topical plaster	95 (79.2)	25 (20.8)	78 (60.9)	50 (39.1)	44 (48.4)	47 (51.6)	.000^∗^
Analgesic tablet	11 (9.2)	109 (90.8)	7 (5.5)	121 (94.5)	20 (22.0)	71 (78.0)	.001^∗^
Acupoint pressure	55 (45.8)	65 (54.2)	77 (60.2)	51 (39.8)	83 (91.2)	8 (8.8)	.000^∗^

∗ Pearson Chi-Squared was used for test, *P* < .01.

### 3.4 The use frequency of 42 commonly used medicinal materials in different regions

**Figure 1:**
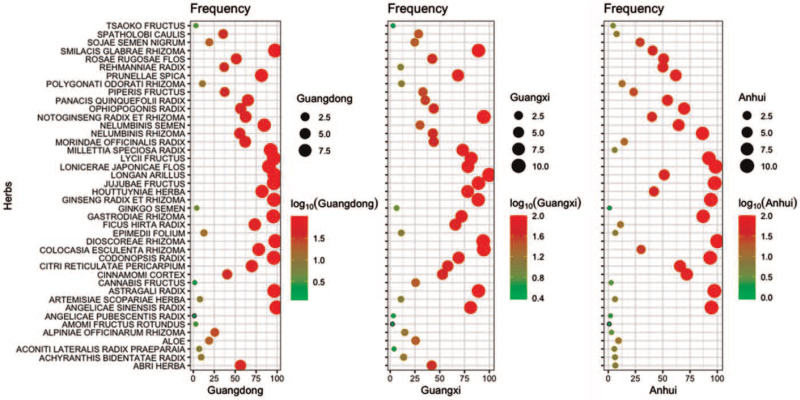
The use frequency of 42 commonly used medicinal materials conducted through questionnaire surveying collegiate athletes in different regions.

**Figure 2:**
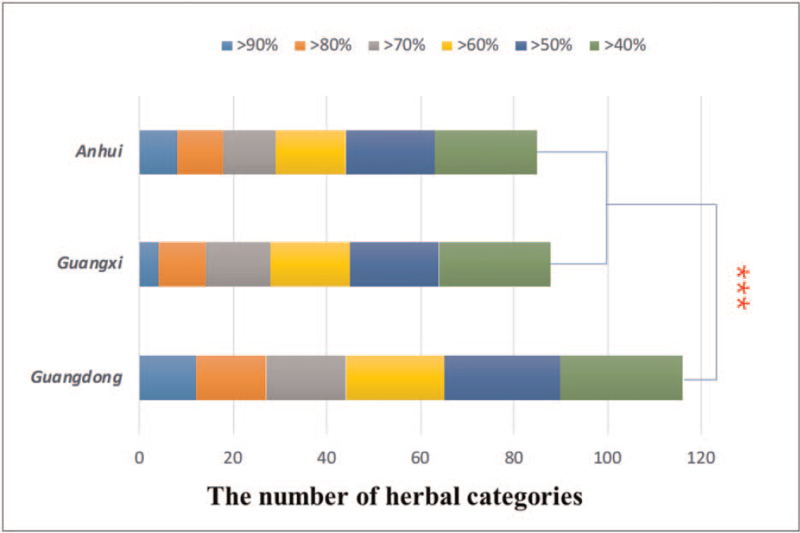
Differences in the number of herbs used by the fixed proportion of colligate athletes in different regions. (^∗∗∗^: *t*-test *P* < .01, N = 3).

The use frequency of 42 commonly medicinal materials used by collegiate athletes was analyzed and showed in the Figure 1, easily showed that the use frequency of herbal medicines is extremely high in Guangdong, compared with Guangxi and Anhui. As shown in Figure 2, the results of scientific statistics also showed that the sorts of herbal medicines consumed by collegiate athletes in Guangdong is significantly higher than that in other places (*t*-test, *P* < .01). As shown in Table 4, the number of medicinal herbs used by the fixed proportion of colligate athletes was also investigated, and the results showed that 21 commonly used medicinal materials are used by 60% of those questioned in Guangdong, while Guangxi and Anhui just have 17 and 15 ones, respectively.

**Table 4:** The number of medicinal herbs used by the fixed proportion of colligate athletes.

Regions	>90%	>80%	>70%	>60%	>50%	>40%
Guangdong	12	15	17	21	25	26
Guangxi	4	10	14	17	19	24
Anhui	8	10	11	15	19	22
Total	24	35	42	53	63	72

## 4 Discussion

China is the country with the largest legal application of traditional Chinese herbs.^[8]^ About 500 kinds of commonly used botanical medicines are routinely equipped in community hospitals, health centers and pharmacies.^[9,10]^ However, the chemical components of each kind of botanical medicines are not fully understand by ordinary sports participants or athletes and even have not been fully isolated and studied by professional researchers. Moreover, as important sources of abundant Chinese food ingredients, herbal materials are appearing in various dishes as flavoring agents or spices.^[11]^

At present, “sports doping” has gradually transitioned from simply referring to substances with sports excitement to the current general terms for all abnormal sports performance improvements.^[12,13]^ So, the principle of entering the sports doping list is based in part on the modern physiological and pharmacological mechanism of action. According to the banned list of international standards in the world anti-doping regulations updated in 2017, all substances or biochemical behaviors that have potential to affect athletes’ sports performances should be covered in the category of the sports doping.^[14]^ It is noteworthy that several plant-derived chemical substances were included in the category of sports stimulants, such as ephedrine, noraconitine, higenamine and morphine. In fact, the potential doping threat from plants far exceeds the estimates of athletes and sports authorities. We investigated the distribution of 13 plant-derived sports stimulants in commonly used herbal materials and the typical application scenarios by collegiate athletes. As shown in the Table 5, the plant-derived substances can be divided into 6 categories including β_2_ receptor agonists, nonspecific stimulants, specific stimulants, anesthetic, cannabis, and other substances listed with surveillances. With our literature verification, the ingestion of more than 300 plant species in 15 categories may trigger the risk of sports doping. It is worth noting that the China Anti-Doping Center issued a special document on the potential risks of noraconitine in November 2016^[14,15]^ “Notice on Strengthening the Prevention and Control of the Positive Risk of Noraconitine,” which involved 7 categories of plants, 47 kinds of Chinese patent medicines or plasters, 2 kinds of cosmetics such as massage lotion and body lotion, as well as 2 kinds of food materials. As shown in Table 5, the results indicated for the first time that more than 7.0% of collegiate athletes in South China use health-care herbs irregularly to trigger the sports restriction of noraconitine. Besides, the most challenging thing is that up to 84.66% of the collegiate athletes have used lotus seeds or lotus plumule for many times, while the lotus seed is a kind of seed food comes from the aquatic plant *Nelumbo nucifera* which has been reported to contain large amounts of norubicine and higenamine, and can directly interfere with cardiovascular function related receptors.

**Table 5:** The classification and potential propensity of athletes’ easy intake of sports doping.

NO.	Category	Compounds	Source plants	FAS^∗^
1	β2 receptor agonists	Noraconitine Higenamine	*Aconitum* plants; *Asarum* plants; some plants in *Annonacea, Gnetaceae, Papaveraceae, Lauraceae.*	5.60%∼7.08%
			***Nelumbo nucifera*** ^∗^	30.09%∼84.66%
2	Nonspecific stimulants	Cocaine	*Erythroxylum*	—
3	Specific stimulants	Ephedrine	All plants in *Ephedraceae*	0.59%∼2.65%
4		Methylephedrine		
5		Pseudoephedrine		
6		Norpseudoephedrine		
7		Levodeoxyephedrine		
8		Strychnine	*Strychnos* plants	—
9	Anesthetic	Morphine	*Papaver* plants	0.88%∼3.24%
10	Other substances listed in monitoring procedure	Codeine		
11		Caffeine	Tea, cocoa, coffee	100.00%
12	Cannabis (cannabinoids)	Tetrahydrocannabinol	*Cannabis sativa*	1.77%∼25.66%
13		Cannabis resin		

∗ FAS = frequency of appearance in this survey.

The analgesic and hallucinogenic substances in Chinese herbal medicines also should not be ignored by athletes and sports management organizations. Due to the widespread use of herbal plasters and topical oils, more analgesic ingredients should be regulated, or at least included in the monitoring list. As shown in Table 3, collegiate athletes in Guangdong province tend to use the topical plasters (79.2%) and herbal oil (45.0%) to deal with their physical stress. As shown in Table 5, our results also first showed that up to 3.24% of the collegiate athletes, who regularly consume the papaveraceae herbs through herbal plasters or Sichuan hot pot; Because of the preference for a special flavor of chicken soup, up to 25.66% of collegiate athletes in Guangxi province regularly consume the hemp seeds, which contains cannabinoids and may lead to serious sports effects. With the exception of a lot of the reported analgesic alkaloids in *Ranunculaceae, Solanaceae,* and *Tetrandridaceae*, there are more and more analgesic and narcotic substances are being found and isolated in an increasing number of plant families and species, which is still unknown to the public, due to the lag of scientific popularization of traditional Chinese medicines.^[15,16]^

In addition, there are nearly 100 kinds of traditional Chinese herbs that have been reported to have potential interactions with receptors related to cardiovascular function and then have the potential to intervene in the sports performance.^[17]^ With the further development of the receptor theory, the interaction of the plant-derived chemicals with cardiovascular-related receptors will be further revealed and disclosed to the general public and special occupational groups. However, at present, there is no clear evidence to prove the correlation between the unrestricted Chinese herbs and the improvement of sports performance. Therefore, we suggest here based on the perspective of sports medicine, that an in-depth study should be developed for explaining the overall effects of the herbal medicines on cardiovascular-related receptors and the corresponding dynamic response of human motor functions.

### 4.1 Limitations

The research results can be generalized only to the Anhui, Guangdong and Guangxi provinces, and because of the wider distribution of 9iological resources and the wider application of herbal medicines, collegiate athletes in Southwest China, including Yunnan, Sichuan and other wider inland areas, may urgently need to carry out a more systematic and comprehensive investigation on the potential risk of the ingestion of traditional herbal materials.

## 5 Conclusion and suggestions

Taken together, this study has first conducted a clearer questionnaire survey on the potential risk of traditional herbal materials ingested by collegiate athletes. It was found that collegiate athletes resorted to some traditional herbal materials to protect their health care that there were regional differences. collegiate athletes with Health Fitness and Traditional Wushu as their sports expertise showed higher recognition and ingestion frequency in the use of traditional herbal materials, while their different living types and cuisine preferences did not seem to be associated with the ingestion frequency of traditional herbal materials. The results also showed that the collegiate athletes with different exercise intensities and different physical diatheses have basically the same purpose of resorting to herbal medicines. The most important thing is that we have found that young athletes do unconsciously intake some kinds of plant-derived sports doping, which may seriously affect their own sports training and competition. In addition, in the view of the significant differences in the use of herbal preparations to relieve sports stress among young athletes in different regions, we strongly suggested that athletes should strictly control their use of various herbal preparations during sports training and competition, including herbal wines, herbal oils, topical plasters, analgesic tablets.

## Acknowledgments

The authors would like to thank Zhuhai Huan’ao Limited for editing and proofreading this article; thanks for the financial support from the special fund for talents in Guangxi University of Traditional Chinese Medicine (2018MA050) and the Key Project of Humanities and Social Science Research in Anhui Province (SK2020A0252).

## Author contributions

**Conceptualization:** Yue-Quan Qin, Ying Jiang, Wei Qi, Bin Zhang, Dong-Yi Wei.

**Data curation:** Yue-Quan Qin, Ying Jiang, Wei Qi, Chunsong CHENG, Dong-Yi Wei.

**Formal analysis:** Ying Jiang, Wei Qi, Bin Zhang, Chunsong CHENG, Dong-Yi Wei.

**Investigation:** Yue-Quan Qin, Bin Zhang, Dong-Yi Wei.

**Methodology:** Yue-Quan Qin, Dong-Yi Wei.

**Software:** Chunsong CHENG.

**Writing – review & editing:** Chunsong CHENG.
